# Mechanical and Thermo-Regulative Investigations on Additively Manufactured Backpack Pads

**DOI:** 10.3390/polym17060738

**Published:** 2025-03-11

**Authors:** Niko Nagengast, Yehuda Weizman, Michael Frisch, Tizian Scharl, Franz Konstantin Fuss

**Affiliations:** 1 Chair of Biomechanics, Faculty of Engineering Science, University of Bayreuth, D-95447 Bayreuth, Germany; yehuda.weizman@uni-bayreuth.de (Y.W.); michael.frisch@uni-bayreuth.de (M.F.); tizian.scharl@uni-bayreuth.de (T.S.); franzkonstantin.fuss@uni-bayreuth.de (F.K.F.); 2 Division of Biomechatronics, Fraunhofer Institute for Manufacturing Engineering and Automation IPA, D-95447 Bayreuth, Germany

**Keywords:** thermo-mechanical recycling, additive manufacturing, polymer characterization, Fused Filament Fabrication (FFF), extrusion-based 3D printing

## Abstract

Backpacks play a pivotal role in facilitating the transportation of essential items, particularly within the realm of physical activities. In demanding physical environments such as mountain sports, effective thermoregulation, pressure absorption, and distribution become paramount due to the repetitive interaction between the athlete’s back and the corresponding area of the backpack. Given that the backpack pads serve as a crucial component of this system, acting as the intermediary layer between the human body and the backpack itself, this study delves into the mechanical and thermoregulatory properties of these components. Specifically, it compares a commercially available pad configuration with five lattice structures manufactured using additive manufacturing techniques. These methods include Large-Volume Filament printing, Multi-Jet Fusion, High-Speed Laser Sintering, and Laser Sintering, with an additional post-processing step—smoothening—for the Multi-Jet Fusion pads. All pads are evaluated on both standardized test protocols regarding mechanics, surface roughness, and humidity as well as a biomechanical setup. For continuous measurement during biomechanical testing, a sensor system including pressure, humidity, and temperature sensors is developed. In addition, a thermal camera was used to measure surface temperature at the back. Throughout the biomechanical testing, 20 male athletes performed a 15 min treadmill walk at 5 km/h and an incline of 6° with all pad configurations, wearing a commercially available backpack with an additional 8 kg of mass. The results revealed significant preferences regarding temperature and humidity uptake, backed up by the standardized test procedures. Furthermore, investigations with the customized sensor system show the irrelevance of the damping-improved back plate design. Overall, additively manufactured backpack pads can play a pivotal role in the thermoregulation and personalized design of backpack configurations.

## 1. Introduction

Backpacks serve as indispensable accessories for many individuals, offering both practical functionality and convenience. They are utilized by various demographics, including students, professionals, military personnel, and athletes. Everyday backpacks prioritize aesthetics, user-friendliness, and utility [1].

Specialized backpacks tailored for outdoor activities such as hiking or operational use must meet specific requirements. These backpacks need to be durable and lightweight, offer ample storage capacity, and ensure comfort during prolonged wear through features like adjustable straps and ventilation systems [2]. Apart from the physical strain of carrying a backpack, discomfort can arise from increased temperatures and moisture accumulation on the back, particularly during activities like hiking. The body generates heat during physical exertion, and efficient thermoregulation is essential for long-term performance and comfort [3]. However, wearing a backpack impedes the body’s natural cooling process, potentially leading to thermal stress, reduced performance, and impaired decision-making. This can be particularly hazardous in outdoor environments where constant environmental awareness is necessary [4,5,6].

While conventional backpacks with foam padding offer good comfort, they contribute to thermoregulation challenges [7,8]. Efforts to enhance temperature regulation in backpacks include research on different materials or construction methods [9,10]. Additive manufacturing presents new opportunities for creating unique shapes, offering potential for innovative padding configurations. Lattice structures, known for their excellent damping and heat dissipation capabilities, could be a promising option to improve backpack design [11,12].

The presence of blocking layers in thermoregulation, especially concerning evaporation and convection in covered areas, presents an opportunity for improvement. Several studies have established the link between different backpack pad designs and their impact on heat and moisture transfer [4,6,9,13,14,15]. Concepts have been developed and tested for backpack padding aligned with evaporation and convection functions, showing the benefits of reduced skin contact area and improved ventilation [4,6,9,16]. Padding variations include full-contact padding (FCP), arched structures with a mesh layer as a back boundary (AML), and novel pad layout designs to enhance ventilation (NLD).

In addition to thermoregulation, mechanical loads and pressure damping on the back significantly influence overall backpack comfort and performance. Since research on the damping behavior of backpack pads is limited, valuable insights can be derived from studies focusing on materials used in backpack cushioning. Different types of plastic polymer foams are frequently used for shock absorption and damping in backpacks [7,8,17,18]. Polymer foams typically have low density and provide effective heat and sound insulation, as well as efficient energy absorption [19]. Timm and Michel [8] utilized a soft foam layer in conjunction with a hard polyethylene sheet, resulting in reduced mean pressure values. Hadid et al. [17] tested backpack straps with a combination of a stiff inner layer and a soft outer shell. Preliminary research suggests that implementing more modern manufacturing methods, such as additive manufacturing, has the potential to enhance the thermoregulatory and mechanical properties of backpack back padding [20].

The manufacturability of complex geometrical lattice structures through additive manufacturing opens up a new realm of padding designs. The versatility of lattice structures [12,21,22,23], particularly their damping properties and benefits for thermoregulation, can play a vital role in backpack pad design. Different lattice types possess distinct properties influenced by the configuration of the cell structure. Nonetheless, the advantages are understood within the context of a comparison with alternative cell structures or solid bodies [24]. There exists a vast array of lattice structures, each possessing unique mechanical and thermodynamic properties [11,23,25]. For backpack padding, energy absorption and thermal management stand out as the paramount parameters. Materials designed for energy absorption must exhibit the capacity to absorb energy while maintaining sufficient rigidity to endure the impact. Extensive research has delved into energy absorption and vibration damping across various structural designs [12,21,22,26,27]. The ability to absorb energy is often considered a notable advantage of lattice structures owing to their capacity for repeated deformation and dispersion of impact load over time, thereby mitigating maximum impact force [28,29].

The investigation of heat dissipation has encompassed diverse structures and materials [12,22,30,31,32]. Struts within these structures facilitate heat dissipation through conductive processes, redistributing heat away from areas of concentrated heat accumulation [32]. This fosters a more uniform heat distribution. The porous and air-permeable nature of lattice structures results in a high surface-to-volume ratio [11,29], rendering them conducive to efficient heat dissipation, given that surface area is pivotal for heat transfer between objects. Structures with open cells exhibit superior convective heat transfer compared to those with closed cells [12,22,23,30,31,32].

**Figure 1:**
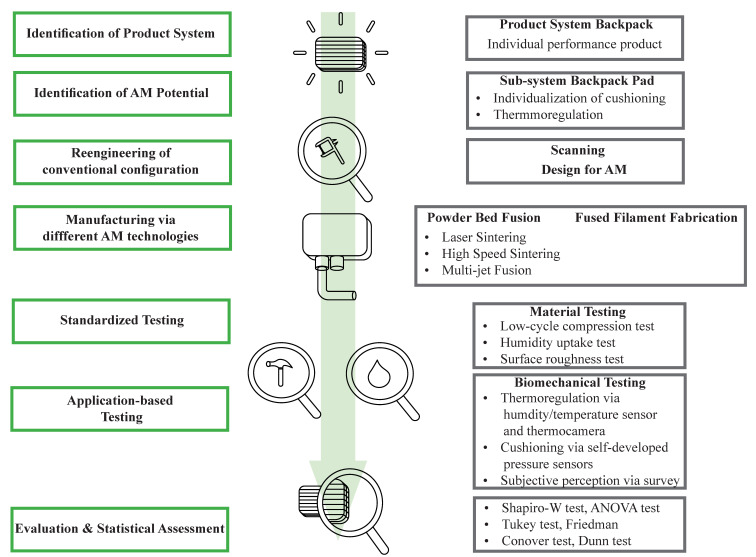
High-level workflow of the proposed study including the identification of the potential benefit of AM, the reengineering and design of an AM substituted part, and the manufacturing, testing, and assessment of it.

This study aims to elaborate on the advantages of additive manufacturing to improve the performance of backpack padding regarding mechanical and thermo-regulative parameters (Figure 1). A total of six different pad designs (one conventional foam-textile pad, one gyroid structure manufactured through Large-Volume Filament printing (Fused Filament Fabrication (FFF) process), one Weaire–Phelan structure manufactured via Multi-Jet Fusion (MJF), High-Speed Sintering (HSS), and Laser Sintering (LS), and one Weaire–Phelan structure manufactured via Multi-Jet Fusion and post-processed (surface smoothening) via sand-blasting) are investigated based on mechanical and thermo-regulative properties. Low-cycle compression, humidity, and surface roughness testing complement the sensor-based measurements throughout biomechanical testing. An application-based sensor system with pressure, humidity, and temperature sensors is developed and implemented into the test setup, complemented by a thermal camera. Furthermore, a survey based on perceived pressure and thermoregulation parameters is performed.

## 2. Materials and Methods

The present research is focused on the reengineering part of the backpack pads, followed by the testing procedures of the single configurations. Furthermore, the setup and methodology of the application-based biomechanical testing is described. Finally, the data processing and statistical analysis are explained.

### 2.1. Design and Manufacturing

**Figure 2:**
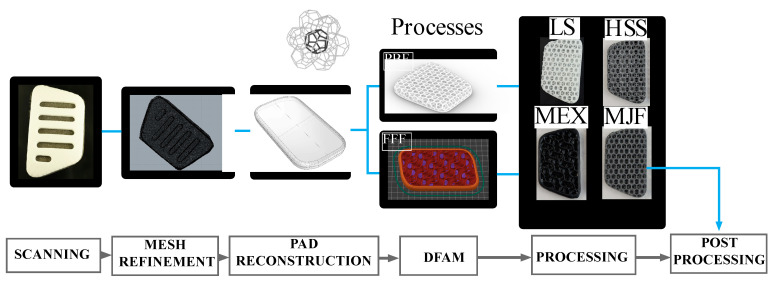
Reengineering and manufacturing workflow with Design for Additive Manufacturing (DFAM) of a commercially available backpack pad (far left) by integrating a Weaire–Phelan structure for the powder-based (PBF) as well as gyroid structure for the filament-based AM procedure(s) (FFF) for the corresponding pads. LS = Laser Sintering, HSS = High-Speed Sintering, MEX = Material extrusion, MJF = Multi-Jet Fusion.

Regarding the redesigning of the backpack pads, a reengineering workflow (Figure 2) of a commercial pad configuration integrated into an Ortovox Traverse 20l backpack (Ortovox, Taufkirchen, Germany) was performed. The standard pads were structured in the following way: a soft EVA foam material facing toward the backpack, a harder EVA foam material on top facing toward the back, and a textile mesh as a cover. For the scanning procedure, the textile mesh was separated. The resulting foam geometry was scanned using the Structure Sensor Pro (Structure, Boulder, CO, USA) integrated into an iPad 11 Pro (Apple, Cupertino, CA, USA), as well as the corresponding scanning software. Mesh clean-up and refinement, as well as pad reconstruction, were performed via Rhinoceros 3D (McNeel, Seattle, WA, USA). The design and tessellation of a Weaire–Phelan cell (20 mm cell size, 1.6 mm strut size) as a common representation of a foam-like microstructure [1] was realized with the Grasshopper Plug-in (McNeel, Seattle, WA, USA). The gyroid structure (35 mm cell size, 2.5 mm surface thickness) of the FFF process was directly implemented using Prusa slicing software 2.6 (Prusa, Prag, Czech Republic).

In total, four different AM manufacturing processes were used (Table 1) to manufacture the backpack pads, by four different companies/institutes. The Weaire–Phelan structure was printed by Rolaserit TPU PB01 powder (AM Polymers, Willich, Germany) via the HSS machine VX 200 (Voxeljet, Friedberg, Germany) and the LS machine 3D Systems Vanguard HS (Vanguard, Philadelphia, PA, USA), as well as by Ultrasint TPU1 powder (BASF, Ludwigshafen, Germany) via the MJF machine HP 5200 (HP, Palo Alto, CA, USA). An AMT PostPro SF (PostPro, Sheffield, UK) was used for smoothening. The gyroid structure was manufactured by Ultrasint TPU1 granulate (BASF, Ludwigshafen, Germany) via Fused Filament Extrusion by the portal printer DX025 (HansWeber, Kronach, Germany) utilizing the extruder AE 20 15, 5D (HansWeber, Kronach, Germany) and a 2 mm nozzle, resulting in MEX pads. The different print parameters are listed in Appendix A, Table A1, Table A2 and Table A3, except for the MJF printing process because the data were not supplied by the company.

### 2.2. Material Testing

**Figure 3:**
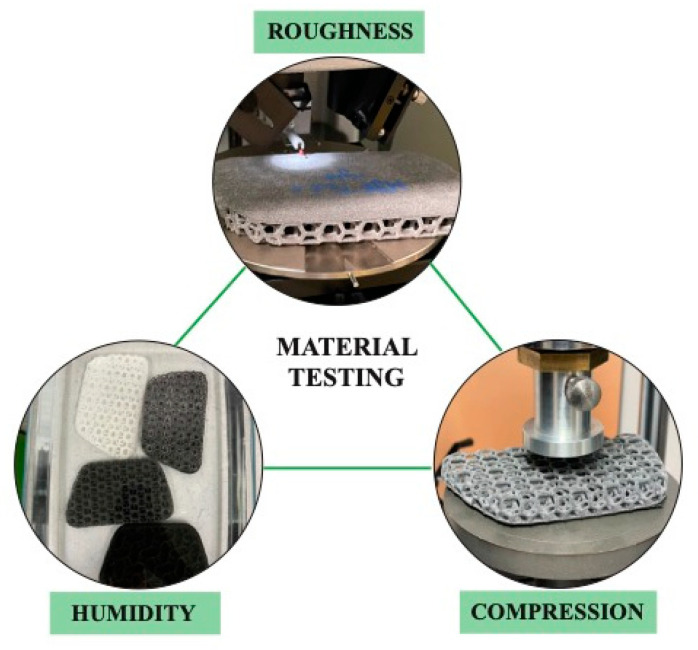
Material testing procedures of the various backpack pads including surface roughness testing, humidity testing via ISO 1817:2024 [33] of all six pad configurations, and additionally, the foam and textile of the standard pad separately, and a low-cycle compression test of all configurations.

The pad configurations were tested (Figure 3) regarding thermo-regulative and cushioning parameters. The standardized humidity and the surface roughness test helped to understand humidity uptake and potential friction-based temperature development, whereas the low-cycle compression test was important in order to set the application-based pressure values in relation.

**Compression testing.** The six pads were indented to a depth of 6–8 mm using a materials testing machine (Z050, ZwickRoell GmbH & Co. KG, Ulm, Germany) and an aluminum piston with rounded edges (Ø 50 mm, Figure 3). Five cycles with a triangular displacement profile were performed at a compression rate of 10 mm/s. The force (F)–displacement (x) curve of the last compression cycle was analyzed, and the following parameters of the loading segment were calculated: energy absorbed (E = ∫ F dx); stiffness (k, dF/dx); maximum of E/F (optimal energy absorption point; [34,35]); and x, F, E, and k at optimal energy absorption point.

**Humidity testing.** A humidity test addressing the behavior of thermoplastic elastomers against liquids was performed according to ISO 1817:2024 [33]. All sample pads (standard, MJF, HSS, LS, FFF, and MJF smooth) were conditioned (DIN EN ISO 291 [36], 23 °C, 50% humidity) for 24 h in a climate oven MKF 115 (Binder, Tuttlingen, Germany). Additionally, the decomposed foam and the textile fabric of the standard pad were tested separately. The results were averaged across three samples. The mass $m_{c}$ was measured after conditioning. Afterward, the samples were put into a water bath (fully covered) for 24 h, drained of the water, and measured regarding the uptake mass $m_{u}$. The relative change in mass $∆m_{h}$ regarding the humidity uptake was calculated based on Equation (1).

(1) $$∆m_{h}=\frac{m_{u}-m_{c}}{m_{c}}\;\times \;100$$

**Roughness measurement.** The surface roughness of the pads can potentially explain surface temperature development based on the friction between the subject (body + t-shirt) and the pad. The stylus profilometer Dektak 150 Surface Profiler (Billerica, MA, USA) was used to perform surface characterization. A total area of 5000 μm × 5000 μm of each pad configuration was measured at the plane part of the pad. Ten rows of that area were scoped using the profiler. The standard pad configuration was neglected as the textile mesh structure caused problems with the tactile measurement method of the pin. The average surface roughness $R_{a}$ was calculated via Equation (2) based on the measured height values $y_{i}$ along a pre-defined profile length L over a squared sample area (Figure 3).

(2) $$R_{a}=\frac{1}{L}\int_{0}^{L}|y|dx$$

### 2.3. Biomechanical Testing

For the application-based testing, an outdoor backpack Traverse 20l from the company Ortovox (Thaufkirchen, Germany) was modified to allow simple integration of the sensors, as well as the removal and attachment of the pad configurations. Twenty male subjects (Anthropometric data in Appendix A, Table A4) fulfilled a standardized test protocol with all pad configurations including a survey at the end of each test cycle. The body mass and the body height of the participants were normally distributed (Kolmogorov–Smirnov test, *p* = 0.9301 and 0.7636, respectively) with a symmetrical shape of the distribution (skewness of 0.336 [*p* = 0.512] and –0.2697 [*p* = 0.598], respectively). A detailed description of the backpack instrumentation and the test procedure is explained subsequently.

**Figure 4:**
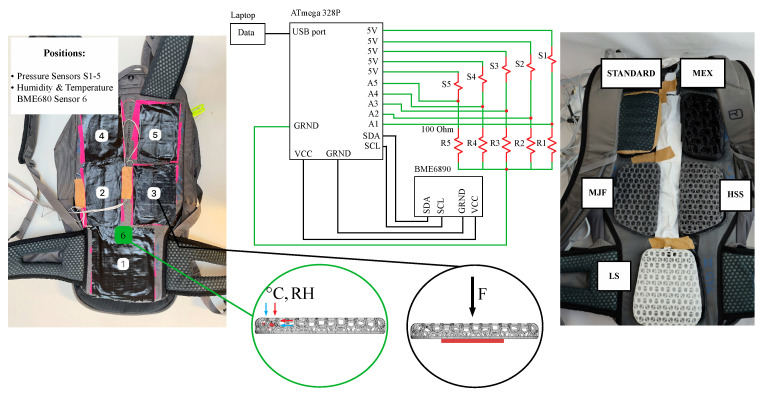
(Left to right) Modified backpack showing the sensor locations (1–5) and the integration of the humidity and temperature sensor (6) inside the lower pad configuration. Circuit diagram of the customized sensor system including 5 pressure sensors, the humidity and temperature sensor, the microcontroller, and the laptop. All 6 pad configurations (standard = standard pad; HSS = High-Speed Sintering; LS = Laser Sintering; MJF = Multi-Jet Fusion; MJF smooth = Multi-Jet Fusion smooth; MEX = Material extrusion) attached to the backpack.

**Backpack configuration**. The quantitative measurement of pressure, temperature, and humidity was implemented by integrating a custom-made measurement system (Figure 4). The standard pads were removed, and five Velostat piezoresistive pressure sensors (3M, Saint Paul, MN, USA) were attached to the back of the backpack exactly where the pads were located. The bottom of the pads and the outside of the sensors were covered with Velcro to allow the quick replacement of the different pad configurations. The humidity and temperature sensors were placed inside the pads (Figure 4) at the location of the highest assumed temperature and humidity uptake.

Before the five pressure sensors were installed in the backpack, they were individually calibrated by placing four masses of 1.25 kg, 2.5 kg, 3.75 kg, and 5 kg on each sensor for 30 s and converting the drop voltage across the reference resistor of the voltage divider to conductance. The function of the logarithm of the gravitational force (N) of the four masses vs. the conductance (mS) was linear and used to create the calibration function of each sensor.

**Figure A1:**
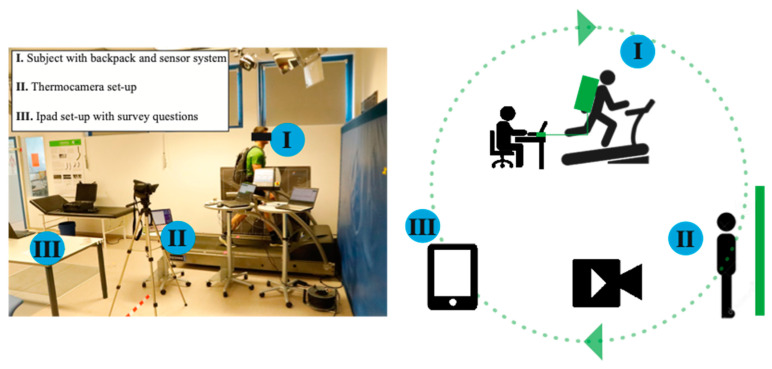
Biomechanical test-set up.

**Test setup**. The biomechanical testing (Appendix A, Figure A1) was performed under laboratory conditions (23 °C and 50% humidity). Each subject was provided with a new polyester t-shirt Puez Sport Dry from the company Salewa (Bolzano, Italy) for each pad configuration ranging in size from L to XL depending on the anthropometry of the subject. Shorts and shoes were chosen individually depending on the subject’s choice. The backpack was filled with an 8 L waterbag from the company Ortlieb (Heilsbronn, Germany). A Variocam (Infratec, Dresden, Germany) thermo-camera was positioned at a standardized distance in front of a blue soft floor mat with markers for the subjects to allow equal positioning. The survey was provided via the survey software Limesurvey 6.1 (Hamburg, Germany) on an Ipad Pro 10th Generation (Cubertino, CA, USA). A treadmill quasar (h/p Cosmos, Nussdorf-Traunstein, Germany) with an adjustable incline was used to execute the test protocol.

**Test protocol**. After the explanation of the test setup and the informative education, the anthropometric data of the subjects were collected. A recording of the back without a shirt was taken with the thermo-camera before any exercise. Following a warm-up phase (10 min; 6° incline, 5 km/h), the thermo-camera recording was repeated. The chronological setup of the six different pad configurations was randomized throughout the 20 subjects. The pad configuration was hidden from the subject. Before each test, all 5 pads, as well as the shirts, were weighed three times and the average value was taken. Each subject was allowed to choose their own fitting of the backpack with the modification of the shoulder and waist straps before the first test execution. The chosen setup remained unchanged throughout the whole test. A walking procedure of t = 15 min, v = 5 km/h, and an incline of 6° was performed for each of the six pad configurations. The sampling frequency of the pressure sensors, as well as the humidity and temperature sensors, was 11 Hz. After each test configuration, a thermo-camera recording of the back without a t-shirt was taken, and the 5 pads and t-shirt were weighted as they were at the start of the test. During the 10 min break between each test, the subject was asked to fill out the survey (Appendix A, Table A5) regarding their perception of the tested pad configuration using a Visual Analogue Scale (VAS).

### 2.4. Data Processing

In this section, the processing of the humidity and temperature sensor data and the thermo-camera data are explained. Furthermore, the calibration of the pressure sensors and the data processing of the pressure sensor data are described.

**Thermoregulation sensor.** In terms of temperature development, two parameters are of interest. The first one is the temperature difference ∆T resulting (Equation (3)) from the pre-test temperature $T_{start}$ and the post-test temperature $T_{end}$.

(3) $$∆T=T_{end}-T_{start}$$

The second temperature-related parameter is the maximum temperature $T_{max}$ measured throughout the test sequence.

Analog to the temperature sensor, the humidity difference ∆H is calculated (Equation (4)) based on the pre-test humidity $H_{start}$ and the post-test humidity $H_{end}$.

(4) $$∆H=H_{end}-H_{start}$$

The second humidity-related parameter is the maximum humidity uptake $H_{max}$ of the thermosensor measured throughout the test sequence.

In terms of statistical significance, a single-factored ANOVA test was performed. If significance was evident, a Tukey–Kramer test was applied.

**Figure 5:**
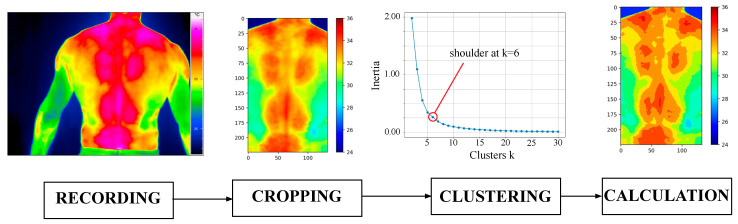
Data processing workflow of the thermo-camera recording.

**Thermoregulation—thermo-camera.** The thermo-camera recordings in the form of an ASCII file of the temperature data were transferred to Python (v.3.14) using OpenCV library for image calculation tasks (Figure 5). The temperature data are represented as an array containing the temperature values of each pixel. Each picture was cropped vertically based on the lower end of the cervical spine and the lumbar spine and horizontally based on the hip width. A cluster count of 10 was selected based on the elbow method [37]. Segmentation into the four regions (shoulder, upper pad, middle pad, and lower pad) was performed. The temperature $T_{seg}$ of each segment was calculated (Equation (5)) based on the sum of the temperature $T_{pixel}$ of the corresponding pixel and the amount *n* of pixels.

(5) $$T_{seg}=\frac{1}{n}\sum T_{pixel}$$

The comparably low temperature of the soft mat was filtered out of the arrays. The surface temperature difference ${∆T}_{surf}$ is based on (Equation (6)) the post-test surface temperature $T_{surf\_end}$ and the pre-test surface temperature $T_{surf\_start}$.

(6) $${∆T}_{surf}=T_{surf\_end}-T_{surf\_start}$$

All data were tested regarding a normal distribution (Shapiro–Wilk test) with a significance level of 0.05. Correspondingly, either a one-way ANOVA or the Friedman rank sum test for multiple correlated samples test was used to identify the significantly different groups in the normal or non-normal distributed data. If significant, a Tukey test was applied to the normally distributed data, as well as Dunn–Bonferroni and Covoner (FDR plus FWER) tests for non-normally distributed data with a significance level of 0.05. If 3 out of 4 pairwise comparisons showed a *p*-value smaller than 0.05, a significant difference was assumed.

**Figure 6:**
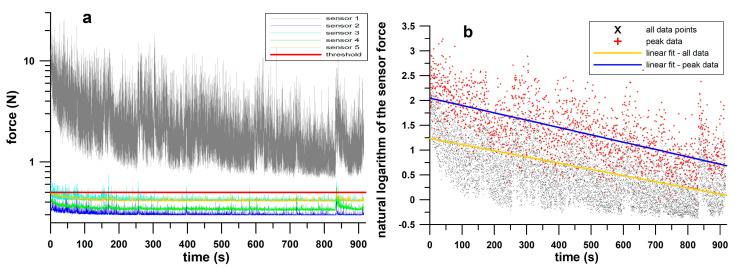
(**a**) Data sample of one participant (sensor force vs. time); (**b**) same sample (natural logarithm of all data and peak data) with linear fits; the slope of the two fits equals the exponential decay constant.

**Force Measurements**. The force (F) data of all five sensors obtained from the calibration curves were examined for a significant sensor response, the threshold of which was the force median of a sensor > 0.5 N (Figure 6a). The force signal of the responding sensors was processed by identifying the peak points, defined by the change in the force rate from positive to negative. For both the entire force dataset and the peak data (Figure 6b), we calculated the medians, the IQR, the skew, and the exponential decay constant. In addition, the kurtosis of the entire force dataset was calculated, as was the difference between the medians of the peak force data and the entire force dataset. To identify the force differences between the 6 pads, we compared the medians of the force parameters using the Friedman rank sum test for multiple correlated samples, and the post hoc Dunn–Bonferroni test was used to identify the significantly different groups.

## 3. Results

### 3.1. Mechanical Testing

The results of the mechanical tests are shown in Table 2. The best energy-absorbing structure was LS with a maximum E/F of 0.0048, followed by MJF with 0.0045. The worst energy absorption structures were the standard pads and MEX with a maximum E/F of 0.0031 and 0.0032, respectively. Interestingly, the latter two structures had the smallest and largest force, stiffness, and energy absorption at the optimal energy absorption point. Since all five 3D-printed pads had the same thickness, LS had the maximum deflection and HSS had the lowest.

### 3.2. Force Measurements

During the treadmill tests, only one sensor responded (sensor) 1, which was attached to the participants’ sacrum. The load of the four other sensors was, on average (median), less than 0.5 N.

The medians of all data per experiment ranged from 0.75 to 2.06 N and the medians of the peak data ranged from 0.82 to 4.64 N across all pads.

When comparing the medians of the force parameters of all six pads using the Friedman rank sum test, the Friedman *p*-value was >0.05 for the skewness of all and peak data, as well as for the kurtosis of the entire force dataset. For the remaining parameters (medians, IQR, and exponential decay constant (EDC) of the peak force data and the entire force dataset, and the difference between the medians of peak forces and all forces ΔF), the Friedman *p*-values were <0.05, and the Dunn–Bonferroni test identified significant differences between the following groups:

- 20 significant differences between the standard pads and the 3D-printed pads, 12 of which were between the standard pads and the LS and MJF pads (6 each). Standard pads generally had larger forces, larger IQR, larger EDC, and larger ΔF.
- Only 3 significant differences between MEX pads and HSS, LS, and MJF-smooth pads, with ΔF being smaller for MEX pads.

**Figure 7:**
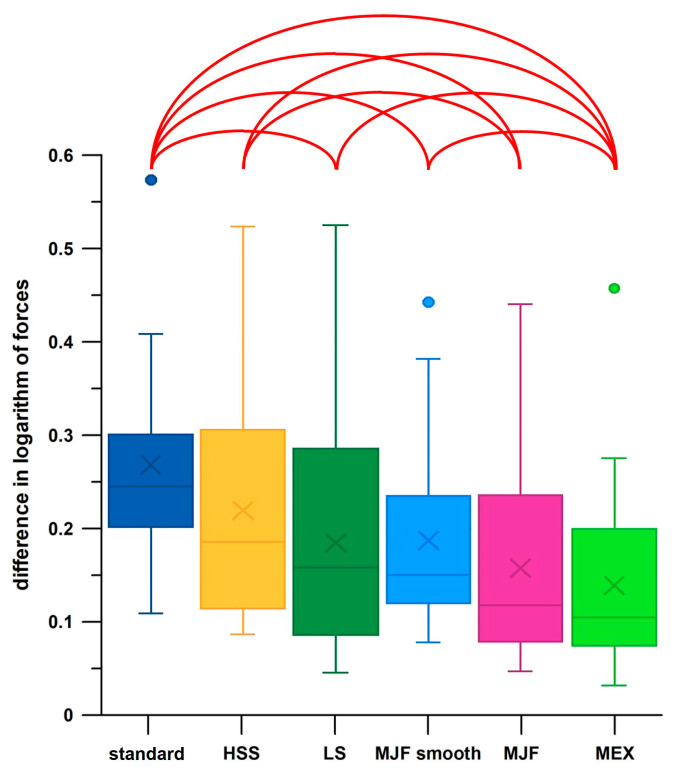
Differences in decadic logarithms of the medians of peak and all forces; standard = standard pad; HSS = High-Speed Sintering; LS = Laser Sintering; MJF = Multi-Jet Fusion; MJF smooth = Multi-Jet Fusion smooth; MEX = Material extrusion; the red arches indicate significant differences; ● = outliers.

The most significant differences (eight) occurred in ΔF (Figure 7), with ΔF being the largest for standard pads and smallest for MEX pads.

When comparing the parameters of the entire datasets to those of the peak force data using the Wilcoxon signed rank test, significant values were found in four parameters. The average medians of the peak data were significantly higher than those of all data, as expected. In addition, the average IQR and exponential decay constants were significantly higher in the peak data, and the peak data were less skewed than the entire dataset.

### 3.3. Humidity Testing

**Figure 8:**
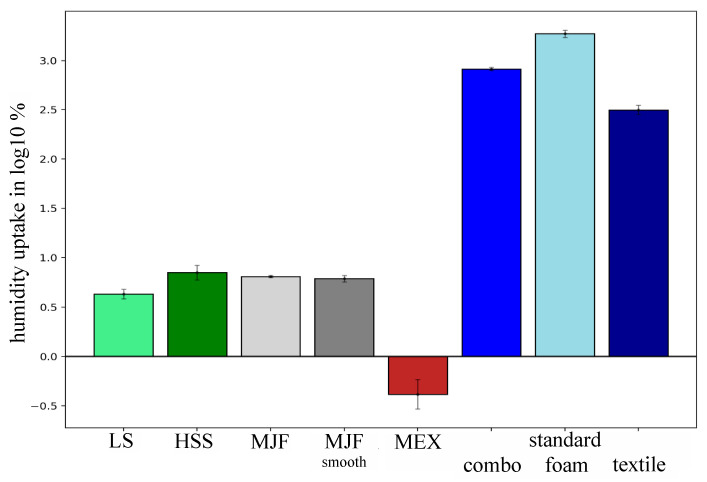
Bar chart of the humidity uptake on a log10 scale following ISO 1817:2024 [33] for standardized humidity testing of elastomers and DIN EN ISO 291 [36] for pre-conditioning; standard = standard pad divided into combo = combined, foam = EVA foam only, textile = textile mesh only, HSS = High-Speed Sintering; LS = Laser Sintering; MJF = Multi-Jet Fusion; MJF smooth = Multi-Jet Fusion smooth; MEX = Material extrusion. The negative humidity uptake of the MEX pad results of the logarithmic operation.

A standardized humidity test was performed on all six pad configurations (LS, HSS, MJF, MJF smooth, MEX, and standard) and the decomposed standard pad parts (textile mesh and foam core) following DIN ISO 1817 [33] and DIN EN ISO 291 [36]. The results are displayed on a log10 scale in Figure 8. The additively manufactured pads showed low humidity uptake ranging from 0.44 ± 0.2% (MEX) to 7.14 ± 1.53% (HSS), respectively. The humidity of the standard pad increased by 820.43 ± 37.43% with an increase of 317.69 ± 39.14% and 1874.96 ± 189.43% regarding the textile mesh and the foam core when tested separately.

### 3.4. Surface Characterization

The surface roughness of the additively manufactured pad configurations was measured with a tactile surface profiler. The standard pad was excluded due to the difficulty of analyzing the textile mesh with the tactile profiler. The results (Table 3) demonstrate a similar surface roughness of the four additively manufactured pad configurations, ranging from 17.7 µm of the LS pad to 21.8 µm of the MEX. The smoothing process of the MJF pad resulted in a decreased surface roughness of 8.5 µm for the MJF smooth pad. The single filament strand of the FFF printing process showed the lowest surface roughness of 3.4 µm.

### 3.5. Biomechanical Testing

Application-driven testing was executed by simulating a real-life scenario based on a treadmill walk (5 km/h) with a moderate incline (6°). A customized sensor system was implemented to collect mechanical and thermo-regulative data. A survey was filled out following each pad to quantify subjective perception. The results presented are two-fold: the thermo-regulative data and mechanical data. The emphasis regarding the perceptual data was set toward the thermo-regulative aspects.

**Thermoregulation.**

Thermoregulation data are divided into temperature results and humidity results regarding the integrated thermo-sensor, the thermo-camera data, and the survey questions regarding airflow, humidity, temperature, and friction perception. Furthermore, t-shirt and pad mass were measured and displayed.

#### 3.5.1. Humidity Sensor

**Figure 9:**
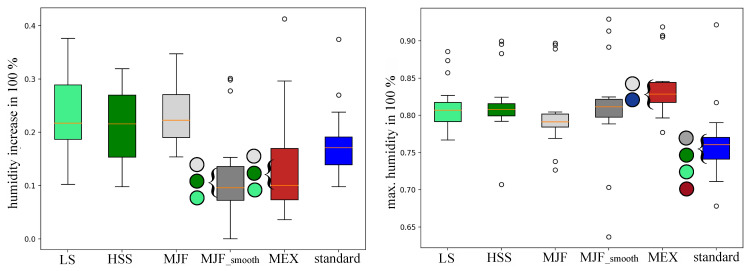
(**left**) Humidity increase and (**right**) maximal humidity between pre- and post-test conditions measured with the implemented thermo-sensor. Colored circles highlight the significant differences between the corresponding backpack pads. Standard = standard pad; HSS = High-Speed Sintering; LS = Laser Sintering; MJF = Multi-Jet Fusion; MJF smooth = Multi-Jet Fusion smooth; MEX = Material extrusion.

Two parameters (Figure 9) were obtained from the customized thermo-sensor regarding humidity: the humidity increase between the start and the end of the test and the maximum humidity after the test. The pad configurations for LS, HSS, MJF, MJF_smooth, MEX, and standard increased by a percentage of 22.97 ± 0.08, 21.36 ± 0.06, 23.40 ± 0.05, 11.88 ± 0.08, 13.59 ± 0.09, and 17.8 ± 0.06, respectively. Both MJF_smooth and MEX showed a significantly (*p*-value < 0.05) smaller increase in humidity uptake regarding the unmodified PBF pads (LS, HSS, and MJF). The maximum humidity of the LS, HSS, MJF, MJF_smooth, MEX, and standard pads showed percentages of 81.03 ± 0.03, 81.40 ± 0.04, 80.00 ± 0.04, 81.09 ± 0.06, 83.56 ± 0.04, and 76.18 ± 0.05, respectively. The standard pad showed a significantly lower maximum humidity than all three PBF pads (LS, HSS, and MJF) and the FFF pad (MEX). On the other hand, the maximum humidity of the MEX pad was significantly higher than the standard and MJF_smooth pads.

#### 3.5.2. Temperature Sensor

**Figure 10:**
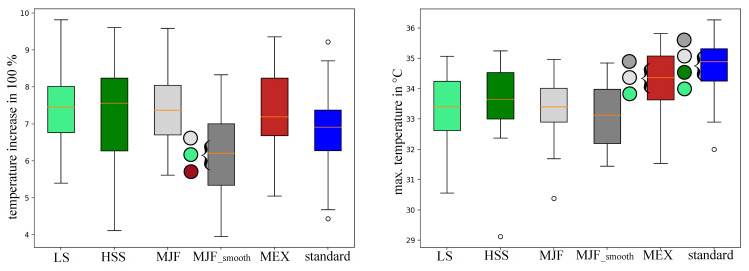
(**left**) Temperature increase and (**right**) maximal temperature between pre- and post-test conditions measured with the implemented thermosensor. Colored circles highlight the significant differences between the corresponding backpack pads. Standard = standard pad; HSS = High-Speed Sintering; LS = Laser Sintering; MJF = Multi-Jet Fusion; MJF smooth = Multi-Jet Fusion smooth; MEX = Material extrusion.

Two parameters (Figure 10) were obtained from the customized thermosensor regarding temperature: the temperature increase between the start and end of the test and the maximum temperature after the test. The pad configurations for LS, HSS, MJF, MJF_smooth, MEX, and standard increased by percentages of 7.46 ± 1.01, 7.25 ± 1.49, 7.39 ± 1.02, 6.17 ± 1.12, 7.32 ± 1.23, and 6.83 ± 1.2, respectively. MJF_smooth showed a significantly (*p*-value < 0.05) smaller temperature increase than the LS, MJF, and MEX pads. The maximum temperature of the LS, HSS, MJF, MJF_smooth, MEX, and standard pad was 33.32 ± 1.14 °C, 33.60 ± 1.35 °C, 33.24 ± 1.05 °C, 33.13 ± 1.06 °C, 34.39 ± 0.98 °C, and 34.73 ± 1.05 °C. In terms of the maximum temperature after the test, the standard and MEX pads showed significantly higher values than the PBF (LS, HSS, MJF, and MJF_smooth) pads.

#### 3.5.3. Survey

**Figure 11:**
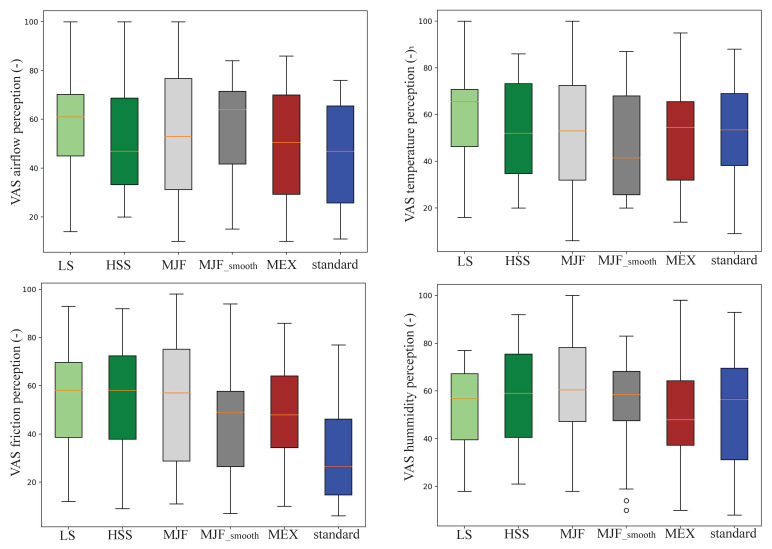
Visual Analogue Score (VAS) of the (**upper left**) airflow perception, (**upper right**) temperature perception, (**lower left**) friction perception, and (**lower right**) humidity perception. The VAS score was taken after each trial of the single pad configurations (LS, HSS, MJF, MJF_smooth, MEX, and standard) throughout a survey representing good/comfortable perception (lower value) and bad/uncomfortable perception (higher value); standard = standard pad; HSS = High-Speed Sintering; LS = Laser Sintering; MJF = Multi-Jet Fusion; MJF smooth = Multi-Jet Fusion smooth; MEX = Material extrusion.

The perception of airflow, temperature, and friction (Figure 11) was addressed throughout the survey questions. The subjects were asked to rate the pad configurations using a Visual Analogue Scale (VAS) from 0 to 100. Regarding the precepted airflow, the LS, HSS, MJF, MJF_smooth, MEX, and standard pads were rated with scores of 57.4 ± 22.3, 51.3 ± 22.6, 54.0 ± 27.5, 56.7 ± 20.1, 49.1 ± 24.3, and 45.3 ± 21.9. The scores for the perceived temperature of the six pads were 58.1 ± 20.86, 53.4 ± 21.0, 53.1 ± 25.3, 48.2 ± 21.6, 51.4 ± 25.4, and 52.5 ± 22.5. Friction was evaluated with VAS scores of 54.4 ± 23.1, 55.4 ± 25.2, 53.8 ± 27.9, 45.3 ± 21.9, 49.1 ± 22.2, and 32.9 ± 22.2. Lastly, perceived humidity was rated with VAS scores of 53.6 ± 17.6, 56.9 ± 20.7, 61.0 ± 24.3, 54.5 ± 20.7, 50.6 ± 23.4, and 53.3 ± 23.4. No significance throughout the single parameters was found.

#### 3.5.4. T-Shirt and Pad Mass

**Figure 12:**
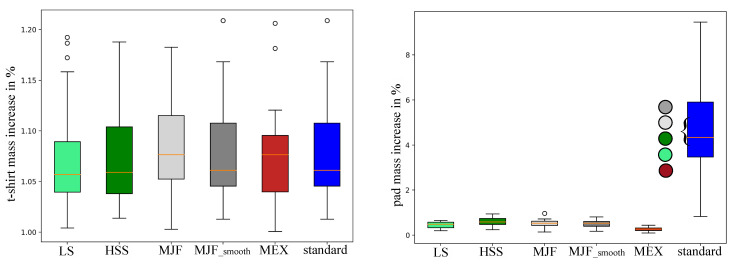
Mass increase between pre- and post-conditions (**left**) of the t-shirt used for each pad condition and (**right**) of each pad configuration. Colored circles highlight the significant differences between the corresponding backpack pads. Standard = standard pad; HSS = High-Speed Sintering; LS = Laser Sintering; MJF = Multi-Jet Fusion; MJF smooth = Multi-Jet Fusion smooth; MEX = Material extrusion.

Each new t-shirt plus the corresponding pad configuration was measured before and after each trial and the results are displayed in Figure 12. The mass of each t-shirt used for each pad configuration increased by 1.07 ± 0.06% for the LS and 1.08 ± 0.05% for all the other pads. No significant difference was observed. The pad mass of the six pad configurations (LS, HSS, MJF, MJF_smooth, MEX, and standard) increased by 0.45 ± 0.14%, 0.59 ± 0.20%, 0.53 ± 0.18%, 0.52 ± 0.16%, 0.26 ± 0.09%, and 4.66 ± 2.08%. The increase for the standard pad was significantly higher compared to all other pads. An overview of the absolute pad weights before and after exposure is summarized in Appendix A, Table A6.

#### 3.5.5. Thermo-Camera

**Figure 13:**
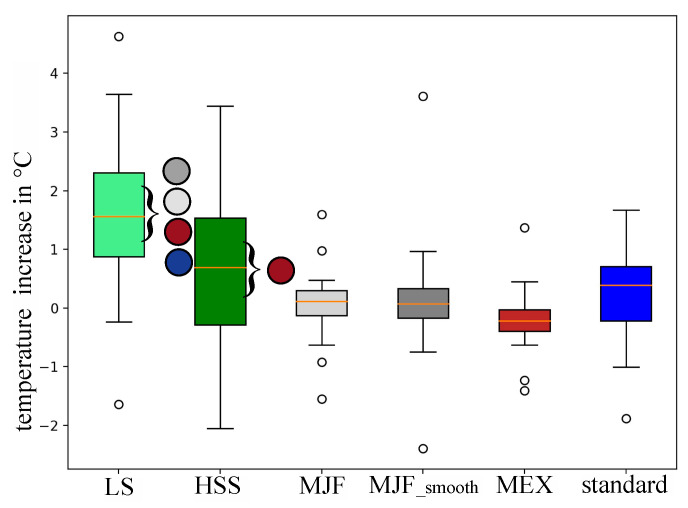
Skin Surface temperature increases between pre- and post-conditions of the investigated pad configurations in the lowest region of the back (see Figure 5). Colored circles highlight the significant differences between the corresponding backpack pads. Standard = standard pad; HSS = High-Speed Sintering; LS = Laser Sintering; MJF = Multi-Jet Fusion; MJF smooth = Multi-Jet Fusion smooth; MEX = Material extrusion.

A thermo-camera was used to investigate skin surface temperature changes between pre- and post-conditions of the different pad configurations. Only the lower back was assessed based on a potential comparison with the integrated temperature sensor in the lowest backpack pad and the biggest contact area between the backpack and back. The temperature increase (Figure 13) of the LS, HSS, MJF, MJF_smooth, MEX, and standard pads was 1.61 ± 1.38 °C, 0.66 ± 1.35 °C, 0.05 ± 0.63 °C, 0.16 ± 1.06 °C, −0.22 ± 0.56 °C, and 0.19 ± 0.83 °C. There was a significant difference in the temperature increase between the LS and MJF, MJF_smooth, MEX, and standard pads, as well as between HSS and MEX pads.

## 4. Discussion

The aim of this work was to investigate thermo-regulative and mechanical behaviors and properties of additively manufactured backpack pads and compare them against the currently available foam-textile composition. Standardized surface characterization and humidity testing complemented the simulation of a real-life application scenario utilizing a backpack for hiking.

The thermo-regulative parameters were measured using a custom sensor in the lower pad. In terms of the measured humidity increase, the values of the MJF_smooth and the MEX pads were significantly lower than the other three AM pads. On the other hand, maximum humidity was significantly higher for the MEX pad and significantly lower for the standard pad. The temperature increase was significantly lower for MJF_smooth in comparison to the LS, MJF, and MEX pads. In consideration of the maximal temperature, significance was found between the MEX and standard pads (higher temperature) in comparison to the PBF pads. The t-shirt mass increased for all pads equally, whereas a significantly higher relative mass increase for the standard pad was shown. No significance regarding subjective perception was found in evaluating the survey results. Skin temperature for the LS and HSS pads also showed significantly higher values, measured by the thermo-camera before and after the trial.

This can be interpreted as follows:

(1) The lower surface roughness (see Table 3) of the smoothed MJF pad and the filament of the MEX decreased friction and therefore increased temperature and humidity development.
(2) The closed structural design of the MEX (closed thermoplastic walls) and standard pad (dense foam) without many airflow options decreased the humidity transport throughout the structure. Furthermore, these chambers can function as traps where heat cannot be transferred efficiently.
(3) The closed structural design of the MEX stores the developed humidity during activity. The composition of the standard pad (textile + foam) allows high storage of humidity in the textile layer. Evidence of the standardized humidity test is shown in Figure 9 and the pad weights in Figure 12, where the mass of the standard pad increased significantly. Therefore, the position of the sensor in the foam core is less exposed to humidity, as long as the humidity uptake of the textile layer is not saturated.
(4) Perception data showed a high deviation, which potentially highlights the wide range of individual preferences. Furthermore, the individual setup of the shoulder belt fixture of each subject and an additional layer between the pad and back can blur exact perceptual statements. Additionally, the sampled subjects do not represent professional athletes with highly detailed perceptions during the performed trial.

The interpreted results have to be put into consideration for the proposed test structure. Despite the low duration (15 min) of physical exposure, significant results are demonstrated. Considering trail and mountain activities that are multiple hours in duration, an upscaling of the effects seems reasonable. The implementation of one thermosensor in this study showed appropriate results in one zone of the thermo-regulative system of the back while carrying a backpack. For future work, it is highly recommended to investigate the whole back and shoulder area to holistically understand heat and humidity dissipation. In terms of the test protocol, the mentioned shoulder-belt fixture can be investigated in more detail to determine the effects in comparison to the presented results. A simple uphill walk (6° incline) was investigated. Further research is suggested to investigate more complicated walking profiles to identify differences in terrain change regarding the thermo-regulative and mechanical properties of a backpack pad. Lastly, hiking or walking only represents one part of the backpack–sports activity interaction. The same test setup should be applied throughout a variety of sports (mountain biking, skiing, and trek running), which could provide more insight into how lattice structures can positively impact and optimize mechanical and thermo-regulative procedures for backpack pad configurations. Finally, this research is thought to be the first comprehensive work of a multi-functional (thermo-regulation/mechanics/perception) assessment of sports components integrating innovative structures and advanced manufacturing methods to push the product development of highly optimized sports products.

Interestingly, the top two rows of symmetrical pads on the back of the backpack were not subjected to any significant force. Only sensor 1 on the sacrum responded. The question raised by this result is whether the other pads are necessary or are rather only a design feature. From a mechanical point of view, a backpack with rigid mass (the water tank in the experiments) has its center of gravity behind the user’s back, creating torque that is balanced by a pulling force at the highest point of the backpack (shoulder straps) and a pushing force at the lowest point (sacrum pads). The same principle can be observed in a climber on a rock face [38]. On the other hand, the mass stored in a backpack is not necessarily evenly distributed, so additional padding makes sense.

Comparing the medians of all measured data per experiment (0.75 to 2.06 N) and the medians of the peak data (0.82 to 4.64 N) of sensor 1 with the optimal force (Table 3) of the mechanical tests, only the force of the standard pads (≈20 N) with which the original backpack was equipped comes closest to the force experienced in sensor 1. However, this only applies to test conditions with an 8 kg mass inserted in the backpack (the water tank in the experiments). All other additively manufactured pads were oversized and therefore not suitable for effective shock and energy absorption. This result is in sharp contrast to the smallest difference between the mean values of the peaks and all data (Figure 7) measured in the MEX pad. The smallest difference means that the peak forces are less pronounced and therefore closer to the median, namely for the hardest pad (MEX; Table 3). There were significant differences between individual groups of the six pads in most of the force parameters derived from the experiments, with the general pattern of the lowest values in the MEX pads and the highest values in the standard pads.

The data from sensor 1 show a clear exponential decay in the measured forces. This decay may have three causes:

1. Participants slightly change their posture during the experiments to accommodate the 8.5 kg ballast mass on their back for load redistribution and pressure relief reasons.
2. The sensor material used in the backpack has a moderate viscosity of 1.24% [39], which is responsible for time-dependent electrical effects such as hysteresis and phase shift [40].
3. The materials of the pads are polymers, which are viscoelastic and therefore susceptible to time-dependent mechanical effects.

Of these three causes, the third is the least likely to occur because the pads do not show a significant difference in the exponential decay constants.

## 5. Conclusions

The present work included the design, manufacturing, standardized, and biomechanical testing of additively manufactured backpack pads with different manufacturing techniques (Laser Sintering, Multi-Jet Fusion, High-Speed Sintering, and Fused Filament Fabrication). These configurations were compared to the commercially available configuration regarding thermo-regulative and mechanical properties. The results showed significant differences regarding thermo-regulative aspects and emphasized the implementation of lattice-based AM structures, especially when post-processed (smoothed) for temperature- and humidity-improved sports apparel. Further outcomes regarding the mechanical properties highlight the differences in cushioning between the different pads but are validated as unnecessary with the outcomes of the biomechanical tests. This study therefore concludes that backpack padding (excluding shoulder-strap padding) serves more as a spacer between the body and the backpack than as a product with improved cushioning, which is especially true during foot-based outdoor activities.

## Figures and Tables

**Table 1:** Investigated backpack pad configurations.

Abbr.	Company/ Institute (City, Country)	Manufacturing Type	Printing Process	Material	Structure	Mass (g)
standard	Ortovox (Taufkirchen, Germany)	Foam extrusion & weaving	-	EVA foam & PE fabric	Open foam & textile mesh	6.1
LS	AM Polymers (Willich, Germany)	Powder bed fusion (PBF)	Laser Sintering	Rolaserit TPU PB01	Weaire-Phelan cell	39.8
HSS	Chair of Remanufacturing (Bayreuth, Germany)	Powder bed fusion (PBF)	Highspeed Sintering	Rolaserit TPU PB01	Weaire-Phelan cell	33.4
MJF	Oechsler (Ansbach, Germany)	Powder bed fusion (PBF)	Multi Jet Fusion	Ultrasint TPU1	Weaire-Phelan cell	31.5
MJF smooth	Oechsler (Ansbach, Germany)	Powder bed fusion (PBF)	Multi Jet Fusion	Ultrasint TPU1	Weaire-Phelan cell	33.1
MEX	HansWeber (Kronach, Germany)	Fused filament extrusion (FFF)	Material extrusion	Ultrasint TPU1	Gyroid cell	59.8

**Table 2:** Energy absorption parameters of the pads; LS = Laser Sintering, MJF = Multi-Jet Fusion, MEX = Material extrusion; MJF smooth = Multi-Jet Fusion smooth, HSS = High-Speed Sintering; x @opt = deflection at the optimum point; F @opt = force at the optimum point; k @opt = stiffness at the optimum point; E @opt = energy at the optimum point; E/F max = maximum ratio of energy to force.

Pad Type	Mechanical Parameters				
	x @opt (mm)	F @opt (N)	k @opt (kN/m)	E @opt (J)	E/F Max (J/kN)
standard	8.0	19.7	6.45	0.06	3.1
LS	8.4	216.0	50.69	1.03	4.8
HSS	6.0	90.0	24.07	0.36	4.0
MJF	8.0	344.7	83.28	1.55	4.5
MJF smooth	8.1	379.7	96.52	1.62	4.3
MEX	6.4	3529.6	1182.45	11.29	3.2

**Table 3:** Measured surface roughness values Ra (µm) of investigated polymers with the corresponding confidence interval (CI95%) of *n* = 3 samples.

	LS	HSS	MJF	MJF Smooth	MEX	MEX Filament
	Quantity = 3, Mean ± std (CI95%)					
**Surface Roughness** **Ra (µm)**	17.7 ± 3.1 (14.2, 21.3)	21.6 ± 1.7 (19.7, 23.6)	18.2 ± 4.6 (13.0, 23.4)	8.5 ± 0.4 (8.1, 9.0)	21.8 ± 1.3 (20.3, 23.3)	3.4 ± 0.3 (3.1, 3.7)

## Data Availability

The data presented in this study are available on request from the first author to any qualified researcher who has obtained Ethics Approval for secondary use of existing data through a Consent Waiver.

## Appendix A

**Table A1:** Printing parameters for MEX pad with DX025 portal printer and AE 20 15,5D extruder.

Parameters	Value
Extrusion width [mm]	2.5
Layer thickness [mm]	1.0
Perimeter [-]	1x
Printing speed [mm/s]	25
Nozzle size [mm]	2.0
Extruder temperature H1–H4 [°C]	175/175/180/180
Printing bed temperature [°C]	70

**Table A2:** Printing parameters for LS pad with 3D Systems Vanguard HS.

Parameters	Value
Layer thickness [mm]	0.1
Process temperature [°C]	100
Fill laser power [W]	38
Fill scan speed [mm/s]	10.180
Fill scan spacing [mm]	0.2
Outline laser power [W]	13
Outline scan speed [mm/s]	1.778
Roller speed [mm/s]	200

**Table A3:** Printing parameters for HSS pad with Voxeljet VX200.

Parameters	Value
Layer thickness [mm]	0.1
Process temperature [°C]	100
Sinter lamp power [%]	100
Grayscale [%]	100
Speed sinter lamp [m/s]	0.1
Speed recoater [m/s]	0.26

**Table A4:** Parameters of the 20 male subjects.

Subject	Mass (kg)	Height (cm)	Shoulder Width (cm)	Acromion Distance (cm)	Shoulder Height—Sitting (cm)	Chest Circumference (cm)
1	78.4	174.0	44.0	35.0	65.5	104.0
2	88.0	180.5	50.0	39.0	73.0	99.0
3	64.6	179.0	42.0	37.0	65.0	89.0
4	94.2	183.5	51.5	39.0	70.0	112.0
5	66.4	180.5	46.0	34.0	66.0	99.0
6	74.6	180.5	45.0	33.0	71.0	96.7
7	81.0	183.0	47.5	34.0	70.0	100.0
8	80.5	181.6	51.0	38.0	72.0	101.0
9	80.9	183.7	48.0	37.0	71.0	98.0
10	85.6	190.0	47.0	38.0	72.0	106.0
11	64.4	168.5	46.0	34.5	65.0	93.5
12	71.2	176.0	45.0	34.5	69.5	99.0
13	81.0	183.0	46.0	39.0	66.0	96.0
14	82.0	186.0	49.0	40.0	72.0	97.0
15	69.0	175.0	49.0	36.0	68.0	96.0
16	90.0	184.0	48.0	36.0	76.0	102.0
17	104.0	191.5	54.0	38.0	78.0	109.0
18	76.8	193.0	49.0	35.0	72.0	94.0
19	57.6	166.0	46.5	31.0	94.5	65.0
20	72.2	173.5	47.0	37.0	68.0	95.0
MEAN	78.1	180.6	47.6	36.3	71.2	97.6
STDEV	11.2	7.0	2.8	2.4	6.5	9.4

**Table A5:** Survey questions with min–max Score.

Nr.	Question	Scoring Interval
1	How do you perceive the wearing comfort of the pad? (0-> bad)	0–100
2	How do you judge the cushioning for the upper pad (0-> no cushioning)	0–100
3	Which value would you perceive as optimum for the upper pad?	0–100
4	How do you judge the cushioning for the middle pad (0-> no cushioning)	0–100
5	Which value would you perceive as optimum for middle pad?	0–100
6	How do you judge the cushioning for the lower pad (0-> no cushioning)	0–100
7	Which value would you perceive as optimum for the lower pad?	0–100
8	How do you perceive the cushioning for the shoulder belt? -> (0-> no cushioning)	0–100
9	Which value would you perceive as optimum for the shoulder belt?	0–100
10	How is the friction for the upper pad? (0-> bad)	0–100
11	How is the friction for the middle pad? (0-> bad)	0–100
12	How is the friction for the lower pad? (0-> bad)	0–100
13	How is the friction for the shoulder belt? (0-> bad)	0–100
14	How well does the upper pad adapt to your back anatomy? (0-> bad)	0–100
15	How well does the middle pad adapt to your back anatomy? (0-> bad)	0–100
16	How well does the lower pad adapt to your back anatomy? (0-> bad)	0–100
17	How comfortable is the temperature at the back by wearing this pad configuration? (0-> comfortable)	0–100
18	How comfortable is the temperature at the lower region of the back by wearing this pad configuration? (0-> low temperature perception)	0–100
19	How well do you perceive the air flow by wearing this pad configuration? (0-> bad)	0–100
20	How well do you perceive the air flow at the lower region of the back by wearing this pad configuration? (0-> bad)	0–100
21	How much humidity development do you perceive by wearing this pad configuration? (0-> no humidity)	0–100
22	How much humidity development do you perceive at the lower part of the back by wearing this pad configuration? (0-> no humidity)	0–100
23	How do you assume your general humidity/sweat production during exercising? (0-> no production)	0–100

**Table A6:** Absolute mass uptake in grams (g) of the various pad configurations by ISO 1817:2024 [33] with the corresponding confidence interval (CI95%) for *n* = 3 samples.

	LS	HSS	MJF	MJF Smooth	MEX	Standard Textile Mesh	Standard Foam
	Quantity = 3, Mean ± std (CI95%)						
Mass before Exposure (g)	39.9 ± 0.2 (39.7, 40.1)	33.2 ± 0.4 (32.7, 33.7)	31.6 ± 0.1 (31.5, 31.7)	33.3 ± 0.2 (33.1, 33.5)	60.2 ± 0.9 (59.2, 61.2)	3.4 ± 0.3 (3.1, 3.7)	2.8 ± 0.3 (2.4, 3.1)
Mass before Exposure (g)	41.7 ± 0.1 (41.6, 41.7)	35.6 ± 0.1 (35.5, 35.6)	33.6 ± 0.1 (33.5, 33.8)	35.3 ± 0.3 (35.0, 35.7)	60.5 ± 0.8 (59.6, 61.4)	14.4 ± 1.0 (13.3, 15.6)	56.3 ± 0.5 (55.7, 56.9)
